# The Bidirectional Relationship Between Cardiovascular Medications and Oral and Gut Microbiome Health: A Comprehensive Review

**DOI:** 10.3390/microorganisms12112246

**Published:** 2024-11-06

**Authors:** Gangani Dharmarathne, Samia Kazi, Shalinie King, Thilini N. Jayasinghe

**Affiliations:** 1 Australian Laboratory Services Global, Water and Hydrographic, Hume, ACT 2620, Australia; 2 Westmead Applied Research Centre, The University of Sydney, Sydney, NSW 2145, Australia; 3 Department of Cardiology, Westmead Hospital, Sydney, NSW 2145, Australia; 4 The Sydney Dental School, Faculty of Medicine and Health, The University of Sydney, Sydney, NSW 2006, Australia; 5 The Charles Perkins Centre, Faculty of Medicine and Health, The University of Sydney, Sydney, NSW 2006, Australia

**Keywords:** CVD, medication, oral and gut microbiome, interaction

## Abstract

Cardiovascular diseases (CVDs) are a leading cause of widespread morbidity and mortality. It has been found that the gut and oral microbiomes differ in individuals with CVDs compared to healthy individuals. Patients with CVDs often require long-term pharmacological interventions. While these medications have been extensively studied for their cardiovascular benefits, emerging research indicates that they may also impact the diversity and composition of the oral and gut microbiomes. However, our understanding of how these factors influence the compositions of the oral and gut microbiomes in individuals remains limited. Studies have shown that statins and beta-blockers, in particular, cause gut and oral microbial dysbiosis, impacting the metabolism and absorption of these medications. These alterations can lead to variations in drug responses, highlighting the need for personalized treatment approaches. The microbiome’s role in drug metabolism and the impact of CVD medications on the microbiome are crucial in understanding these variations. However, there are very few studies in this area, and not all medications have been studied, emphasizing the necessity for further research to conclusively establish cause-and-effect relationships and determine the clinical significance of these interactions. This review will provide evidence of how the oral and gut microbiomes in patients with cardiovascular diseases (CVDs) interact with specific drugs used in CVD treatment.

## 1. Introduction

The term “cardiovascular disease” (CVD) refers to a broad range of illnesses that affect the heart and arteries. It stands out as one of the primary causes of mortality globally; as reported by the World Health Organization (WHO) in 2019, CVDs claimed the lives of 17.9 million individuals worldwide, constituting 32% of all fatalities [1]. From a scientific perspective, CVDs’ causation primarily involves the intricate interplay of multiple factors, including the cardiovascular system, genetics, and lifestyle choices [2]. This category of diseases includes a range of disorders, such as coronary artery disease (CAD), stroke, heart failure, and peripheral arterial disease [2]. CAD, the most prevalent form of cardiovascular illness, develops when the coronary arteries responsible for supplying blood to the heart narrow or become blocked due to the buildup of plaques [3]. This can lead to chest pain or discomfort, medically known as angina [4], or it may cause a complete blockage, resulting in a heart attack. Additional CVDs include heart failure, where the heart’s ability to pump blood efficiently is compromised [5,6]; arrhythmias, which denote abnormal heart rhythms [4,7]; and valvular heart disease, characterized by issues with the heart valves [8,9].

The human microbiome refers to the vast community of microorganisms residing in and on the human body, including their genetic material. This diverse array of microorganisms comprises bacteria, viruses, fungi, and various other microbes [10]. The human microbiome is incredibly diverse, with trillions of microorganisms primarily residing in the gut and oral cavity [11].

The gut microbiome pertains to the microbial population residing in the gastrointestinal tract, encompassing the stomach, small intestine, and large intestine (colon) [12]. It is estimated that there are more than 1000 different species of bacteria in the human gut, and everyone has a unique composition of microbes [13]. These bacteria play vital roles in regulating the immune system and facilitating nutrient absorption and digestion. Additionally, they contribute to the synthesis of essential substances and vitamins (e.g., vitamin K, vitamin B12 and folic acid), as well as other crucial compounds, such as short-chain fatty acids (SCFAs) and essential amino acids (e.g., isoleucine, leucine, and valine), that the human body cannot produce on its own [14].

The oral microbiome, on the other hand, comprises the community of microorganisms that inhabit the mouth and oral cavity, including various species of bacteria, fungi, and viruses [15]. The oral environment is highly diverse and dynamic, influenced by factors such as oral hygiene practices, diet, and overall health [16]. Within the oral microbiome, there exists a balance of both beneficial and harmful bacteria. Some bacteria play a crucial role in maintaining oral health by preventing the overgrowth of harmful species and contributing to the defense against pathogens. However, an imbalance in the oral microbiome can lead to oral diseases such as dental caries (cavities) and periodontal (gum) disease [17].

Interactions between the microbiome and the host play a critical role in shaping human health and have far-reaching implications for the development of various diseases. The microbiome, particularly in the gut, is a complex ecosystem composed of more than 100 trillion (~4 × 10^13^) of microorganisms that coexist with the human host in a mutually beneficial relationship [18,19]. These interactions exert profound effects on numerous aspects of human physiology and contribute to overall well-being [20,21]. However, disruptions or imbalances in the composition of the microbiome, known as dysbiosis, have been associated with a range of diseases. Inflammatory bowel diseases, including Crohn’s disease and ulcerative colitis, have been linked to dysbiosis in the gut microbiome [22]. Other conditions, such as obesity, CVDs, type 2 diabetes, allergies, autoimmune disorders, and certain types of cancer, have also been associated with an altered microbiome composition [23,24].

The relationship between CVD medications and the human microbiome is an area of growing interest. Emerging research indicates that cardiovascular disease medications can influence the microbiome, affecting its abundance and diversity [25,26,27,28,29]. Statins, in particular, have been associated with changes in the gut microbiome, affecting the bacterial abundance and diversity [30,31,32]. While the direct effects of antiplatelet agents on the microbiome require further investigation, emerging evidence suggests potential interactions [33,34].

This review aims to understand how CVD medications interact with the microbiome to uncover insights into their therapeutic effects and aid in developing personalized cardiovascular health management.

## 2. Overview of the Human Microbiome

The development of the human microbiome begins shortly after birth, with a major dose of microbial exposure occurring during delivery, introducing the infant to a significant microbial population for the first time [35,36]. The initial microbial colonization is influenced by several factors. For instance, the method of delivery plays a crucial role, with vaginal births exposing infants to their mother’s vaginal and fecal microbiota, whereas cesarean sections result in different microbial exposure [34]. Exposure to antibiotics during pregnancy, childbirth, or infancy can disrupt microbiome development by altering the bacterial balance [37]. Environmental factors, such as the living conditions and exposure to pets, introduce various microorganisms to infants, further influencing the microbiome’s composition [37,38]. Additionally, the type of feeding, whether breast milk or formula, significantly affects the microbiome composition [13]. These early factors not only shape an individual’s microbiome but also continue to impact its development and stability throughout their life. The gut microbiome undergoes significant changes from birth to age three, becoming more diverse and stable to resemble that of an adult, thereby highlighting the profound link between early life experiences and long-term microbiome health [34,35,36,37].

The oral microbiome undergoes significant changes throughout an individual’s life, adapting to the evolving conditions within the oral cavity and the external environment [37,39]. Common bacteria found in the oral microbiome include *Streptococcus mutans*, *Porphyromonas gingivalis*, *Prevotella melaninogenica*, *Fusobacterium nucleatum*, *Streptococcus salivarius*, and *Veillonella atypica* [40,41]. In contrast, the gut microbiome exhibits greater complexity and diversity. The most prevalent bacterial phyla in the gut include Firmicutes, Bacteroidetes, Proteobacteria, and Actinobacteria [42].

## 3. The Microbiome and CVD

**Figure 1:**
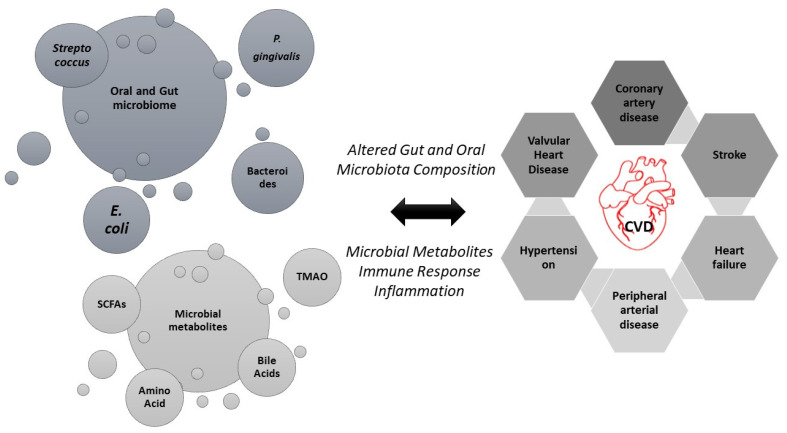
The connection between the altered composition of the gut and oral microbiota and the development of various CVDs. The **left** side of the figure highlights key microbial communities and their metabolites, such as *Streptococcus*, *E. coli*, *P. gingivalis*, Bacteroides, SCFAs, bile acids, and amino acids. These microbes and their metabolites influence the immune response and inflammation, leading to an altered microbiota composition. The **right** side of the figure shows the potential cardiovascular outcomes, including coronary artery disease, stroke, heart failure, peripheral arterial disease, hypertension, and valvular heart disease. The bidirectional arrow indicates the continuous interaction between microbial metabolites, the immune response, and inflammation in shaping cardiovascular health.

Bacterial DNA and viable bacteria in atherosclerotic plaques, which are deposits of fatty substances in the arteries, suggest a possible link between bacterial infections and the development or progression of atherosclerosis [43,44]. The gut and oral microbiomes play a critical role in cardiovascular health, with specific bacterial species and their metabolites being linked to the development and progression of CVDs (Figure 1) [45]. Microbial metabolites, such as trimethylamine N-oxide (TMAO), have been linked to increased cardiovascular risks by influencing cholesterol metabolism and promoting thrombosis [46]. TMAO is produced by gut bacteria during the digestion of certain dietary nutrients, such as choline and carnitine, which are found in foods like red meat, eggs, and dairy products [47]. Certain gut bacteria (*Clostridium* and *Enterobacteriaceae*) metabolize these dietary compounds, such as choline and carnitine, together with phosphatidylcholine and into trimethylamine (TMA), which is subsequently converted to TMAO in the liver [48]. TMAO disrupts cholesterol’s metabolism by increasing the cholesterol deposition in the arterial walls and inhibiting reverse cholesterol transport, both of which contribute to the development of atherosclerosis [49]. Additionally, TMAO enhances platelet reactivity, raising the likelihood of blood clots forming and subsequently increasing the risk of thrombosis [50,51,52]. Gut dysbiosis has also been linked to chronic inflammation, further exacerbating CVDs. Reduced populations of beneficial bacteria such as *Bifidobacterium longum* and *Lactobacillus rhamnosus* can compromise gut barrier integrity, leading to “leaky gut” syndrome [53,54]. This allows endotoxins, such as lipopolysaccharides (LPS) from Gram-negative bacteria like *Escherichia coli*, to enter the bloodstream [54]. Once in circulation, these endotoxins trigger systemic inflammation, contributing to endothelial dysfunction and promoting atherosclerosis [55]. Maintaining a balanced gut microbiome is therefore vital in managing and reducing the risk of cardiovascular diseases.

While the gut microbiome has received more attention in relation to CVDs, there is also ongoing research exploring the potential role of the oral microbiome. Poor oral hygiene and oral diseases, such as periodontal disease, have been associated with an increased risk of CVDs [56]. Roca et al. (2018) found a positive association between periodontal disease and the risk of CVDs, independently of traditional cardiovascular risk factors [57]. Oral bacteria with pathogenic characteristics, especially those linked to periodontal diseases, have the ability to enter the bloodstream [58]. Certain oral bacteria, such as *P. gingivalis*, invade host tissue, evade immune defenses, and trigger chronic inflammation through the activation of Toll-like receptors (TLRs), leading to the release of proinflammatory cytokines such as tumor necrosis factor-α (TNF-α) and IL-6 [59]. Additionally, the bacteria trigger the release of proinflammatory mediators, such as TNF, interleukins (IL-1, IL-6, IL-8), and reactive oxygen species [60]. These mediators, along with microbial by-products, enter the systemic circulation either through swallowing or directly via the blood vessels surrounding the teeth. Once in the bloodstream, they increase systemic inflammation and stimulate the liver to release acute-phase proteins, including C-reactive protein (CRP), pentraxin, and fibrinogen [61]. These proteins, particularly CRP and fibrinogen, contribute to increased blood viscosity, endothelial injury, platelet aggregation, and altered lipid metabolism, all of which elevate the risk of atherosclerosis and thrombus formation [62]. These processes ultimately narrow blood vessels, reducing the blood flow to the heart and other organs, raising the likelihood of cardiovascular complications.

Moreover, *Treponema denticola*, often found in periodontal disease, produces proteases that degrade host tissue and activate immune responses, leading to the increased production of proinflammatory cytokines [63]. These cytokines contribute to endothelial dysfunction [64,65]. *Tannerella forsythia* has been shown to stimulate immune cells, promoting chronic inflammation and contributing to endothelial dysfunction [66]. These bacteria and their toxins, such as lipopolysaccharides (LPS) from *P. gingivalis* or proteases from *T. denticola*, enter the bloodstream, where they directly damage the endothelial lining of blood vessels [66]. This damage impairs the normal function of endothelial cells, which are responsible for maintaining vascular health. Endothelial dysfunction is a critical early event in the formation of atherosclerotic plaques, which can eventually narrow arteries and restrict blood flow [58,67]. Moreover, gum disease (periodontitis) has been linked to endothelial dysfunction, a precursor to atherosclerosis, suggesting a potential pathway through which oral health may impact cardiovascular health [68].

## 4. Cardiovascular Medications and Microbiome Alterations

**Figure 2:**
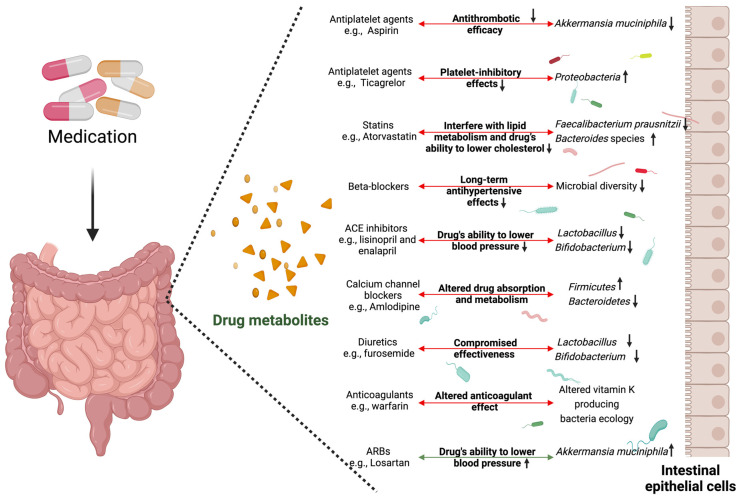
Impact of cardiovascular medications on gut microbiome. Cardiovascular medications, including antiplatelet agents (aspirin, ticagrelor), statins, beta-blockers, angiotensin-converting enzyme inhibitors, angiotensin II receptor blockers, calcium channel blockers, diuretics, and anticoagulants, induce significant changes in the gut and microbiota. These alterations occur through mechanisms that may include direct antibacterial effects, selective pressures, and indirect influences on the gut environment, such as changes in pH, immune modulation, and bile acid composition. These shifts in the microbiome can affect the microbial balance, drug efficacy, and patient outcomes by influencing gut permeability, systemic inflammation, drug metabolism, and side effects. The figure highlights key microbial changes with their corresponding impacts on drug efficacy and disease management (Arrow up: Increasing, Arrow down: Decreasing).

Cardiovascular disease continues to be a leading cause of death, and the management of this condition heavily relies on pharmacological interventions. The selection of specific medications is intricately influenced by factors such as the type and severity of the CVD, individual patient characteristics, risk assessments, efficacy, safety profiles, patient preferences, and adherence. The medications used in the treatment of CVDs can be categorized into different groups based on their specific roles in CVD management, as shown in Figure 2 and Table 1.

Cardiovascular medications can induce significant shifts in the gut and oral microbiota, impacting not only the microbial balance but also drug efficacy and patient outcomes. For example, antiplatelet agents like aspirin are known to reduce beneficial bacteria such as *Akkermansia muciniphila*, which plays a key role in maintaining gut barrier integrity [69]. A reduction in this bacterium can lead to increased gut permeability, promoting systemic inflammation and potentially reducing aspirin’s antithrombotic efficacy [25]. While aspirin does not exhibit direct antibacterial effects, its impacts on the gut pH and immune response may selectively affect certain microbial populations [70]. Similarly, Ticagrelor, another antiplatelet drug, increases the abundance of *Proteobacteria*, a bacterial group associated with inflammation, which may undermine its platelet-inhibitory effects [71]. Statins like atorvastatin have been shown to decrease *Faecalibacterium prausnitzii*, a key anti-inflammatory microbe, while increasing *Bacteroides* species, a change that could interfere with lipid metabolism, possibly reducing the drug’s ability to lower cholesterol effectively and increasing the risk of metabolic side effects [72]. These effects are primarily indirect, arising from the modulation of bile acid metabolism and lipid profiles, which can create an environment more favorable to certain bacterial groups over others [72]. Moreover, studies have shown that statins can reduce the growth of specific bacteria, like *Helicobacter pylori* and other pathogenic strains, due to their ability to interfere with cell membranes [73]. This effect could indirectly disrupt the gut microbial balance, leading to the reduced diversity or loss of certain beneficial species.

In addition, beta-blockers and angiotensin-converting enzyme (ACE) inhibitors also alter the microbial landscape. Beta-blockers tend to decrease the overall microbial diversity, potentially leading to a less resilient gut microbiota, which may diminish their long-term antihypertensive effects [74]. Some beta-blockers, like propranolol, have shown slight antibacterial effects against specific strains such as *E. coli* [75]. These effects can still influence the bacterial composition by reducing certain populations, which could indirectly affect gut and metabolic health. ACE inhibitors, like lisinopril and enalapril, reduce beneficial bacteria such as *Lactobacillus* and *Bifidobacterium*, promoting gut dysbiosis and inflammation, which can impair gut health and affect blood pressure regulation [76,77]. While not directly bactericidal, ACE inhibitors may influence the microbiota composition by impacting intestinal immune responses and gut permeability [76]. These microbial changes can not only affect how drugs are metabolized but also influence patients’ susceptibility to side effects, highlighting the importance of considering the microbiome composition in optimizing cardiovascular treatment strategies.

Moreover, angiotensin II receptor blockers (ARBs) like losartan are known to boost the levels of *A. muciniphila*, a beneficial bacterium that strengthens the gut barrier and enhances the drug’s ability to lower blood pressure [77,78]. ARBs exert their influence primarily by enhancing gut barrier function, indirectly favoring the growth of *A. muciniphila* and similar bacteria [79]. On the other hand, calcium channel blockers such as amlodipine tend to shift the gut microbial balance by increasing *Firmicutes* and reducing *Bacteroidetes*, potentially causing gastrointestinal disturbances and altering how these medications are absorbed and metabolized [80]. Diuretics, like furosemide, can lead to a reduction in beneficial bacteria such as *Lactobacillus* and *Bifidobacterium*, which may result in increased gut permeability and inflammation [81,82]. This shift likely occurs through changes in the electrolyte and water balance in the gut environment, affecting bacterial populations indirectly [82]. This can compromise the diuretic’s effectiveness in managing fluid retention and heart failure symptoms [83]. Additionally, anticoagulants like warfarin can interfere with the gut’s production of vitamin K by altering the bacteria involved in its synthesis, potentially affecting blood clotting and increasing the risk of bleeding or thrombotic complications [84,85]. These interactions highlight the complex relationship between cardiovascular medications and the microbiome, underscoring the need for personalized treatment strategies that account for individual microbiota variations.

**Table 1:** Various medications used in CVD treatment and their specific roles.

Group	Drug Types	Specific Role	Microbiome Alterations	Reference
Antiplatelet agents	Aspirin (Acetylsalicylic Acid), Clopidogrel, Ticagrelor, Prasugrel	Reduce the risk of thrombus formation by preventing the aggregation of platelets and inhibit platelet aggregation.	Gut: Reduce beneficial bacteria like *Akkermansia muciniphila* and increase Proteobacteria (Ticagrelor). Indirectly affect microbiota through gut immune modulation.	[69,86,87,88]
Statins (HMG-CoA reductase inhibitors)	Atorvastatin, Simvastatin, Rosuvastatin, Lovastatin	By lowering LDL-C (improving the lipid profile), it decreases the risk of cardiovascular events such as acute coronary syndromes and stroke.	Gut: Decrease *Faecalibacterium prausnitzii* and increase Bacteroides. Indirect alterations through bile acid metabolism. Oral: Reduction in periodontal pathogens such as *P. gingivalis.*	[72,89,90,91]
Beta-blockers	Metoprolol, Atenolol, Propranolol, Bisoprolol	Lower heart rate and blood pressure, decrease the force of contraction (negative ionotropic response), and manage conditions such as hypertension, angina, and arrhythmias.	Gut: Decrease gut microbial diversity indirectly. Oral: Alterations in the composition of the oral microbiome in individuals with periodontitis.	[74,92,93,94,95]
Angiotensin-converting enzyme (ACE) inhibitors	Lisinopril, Enalapril, Ramipril, Captopril	Lower blood pressure, reduce the strain on the heart, and block the production of angiotensin II. Inhibit the renin angiotensin aldosterone pathway (RAAS), resulting in hypotension and preventing cardiac remodeling.	Gut: Reduce *Lactobacillus* and *Bifidobacterium* abundance.	[76,77,96,97]
Angiotensin II receptor blockers (ARBs)	Losartan, Valsartan, Olmesartan, Candesartan	Reduce blood pressure by blocking the action of angiotensin II on blood vessels and improve the heart’s pumping ability. Inhibit the RAAS, resulting in hypotension and preventing cardiac remodeling.	Gut: Increase *A. muciniphila.*	[77,78,98,99,100]
Calcium channel blockers	Amlodipine, Diltiazem, Verapamil, Nifedipine	Reduce blood pressure by relaxing and dilating the arterial walls, improve blood supply to the heart, and reduce the myocardial oxygen demand.	Gut: Increase *Firmicutes* and decrease *Bacteroidetes.*	[80,101]
Diuretics	Hydrochlorothiazide, Furosemide, Chlorthalidone, Spironolactone	By increasing the production of urine, diuretics work on different components of the renal tract to remove excess fluid, reducing the cardiac preload and relieving symptoms of heart failure.	Gut: Reduce *Lactobacillus* and *Bifidobacterium* abundance.	[81,102]
Nitroglycerin and nitrates	Nitroglycerin, Isosorbide dinitrate, Isosorbide Mononitrate	Provide rapid relief from angina attacks, reduce chest pain, and improve blood flow to the heart during critical situations.		[103,104]
Anticoagulants	Warfarin, Apixaban, Rivaroxaban, Edoxaban	Prevent cardioembolic phenomena including strokes in high-risk conditions such as atrial fibrillation or mitral stenosis.	Gut: Affect vitamin K-producing bacteria.	[84,85,105,106]
Antiarrhythmics	Amiodarone, Flecainide, Propafenone, Sotalol	Restore the heart’s normal rhythm in patients with arrhythmias and alleviate symptoms associated with rapid and irregular heart rates.		[107,108]

## 5. The Role of the Microbiome in the CVD Medication Response

**Figure 3:**
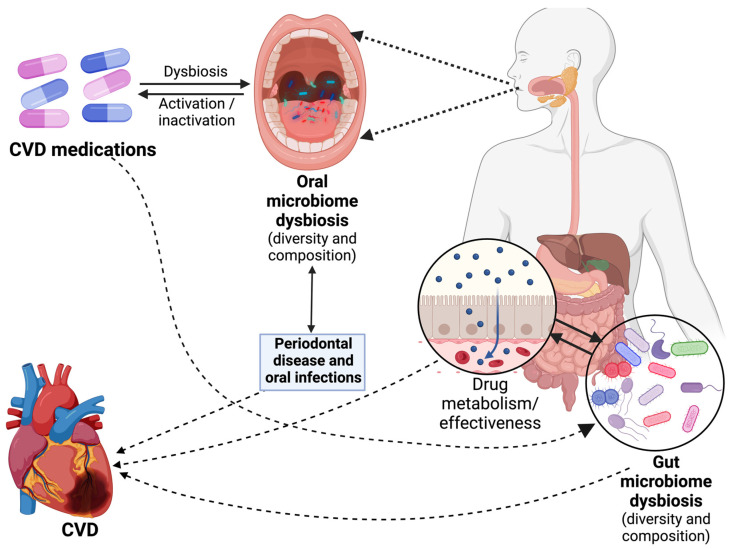
A schematic representation of the potential interactions between the microbiome and cardiovascular disease (CVD) medications, highlighting two key aspects of microbiome involvement: the oral microbiome and the gut microbiome. The left side of the figure zooms in on the oral microbiome, emphasizing its significance in the activation and inactivation of CVD drugs. Dysbiosis within the oral microbiome can impact these processes, potentially affecting the efficacy of medications used in CVD treatment. Moving to the right side of the figure, we shift our focus to the gut microbiome. Here, we illustrate how the gut microbiome also plays a pivotal role in the absorption and metabolism of CVD drugs. Dysbiosis within the gut microbiome also can significantly influence these processes, ultimately affecting the effectiveness of CVD medications.

Individual patients exhibit significant variability in their responses to medications, and adverse events related to drug use contribute substantially to morbidity and mortality [109,110]. This has sparked strong interest in comprehending the host and environmental factors that underline variations in drug responses and the occurrence of adverse events [111,112]. These factors include age, sex, nutritional status, and disease states, as well as genetic and environmental exposures, all of which collectively influence how individuals respond to drug therapies [111,112]. Increasingly, microbiomes are gaining recognition as an often-overlooked contributor to the diversity in drug metabolism and pharmacological efficacy (Figure 3). In particular, the gut microbiome is postulated to influence the response to cardiovascular drugs through several mechanisms, although the precise nature of these interactions is still a subject of ongoing research.

Certain gut microbiomes have been identified as key players in the metabolism of cardiovascular drugs, impacting their efficacy and safety. For instance, *Eggerthella lenta* is known to inactivate digoxin, a heart medication used to treat heart failure, by reducing it into less active forms [113]. This microbial interaction can diminish the drug’s therapeutic effects, leading to suboptimal outcomes [114]. Similarly, *Bacteroides* species are involved in the metabolism of statins, commonly used to lower cholesterol levels. These bacteria can modulate statins’ bioavailability, affecting the drug’s cholesterol-lowering efficacy [115,116]. Another example is *A. muciniphila*, which can enhance the efficacy of certain antihypertensive drugs by improving drug absorption [117]. Additionally, *Clostridium* and *Lactobacillus* species have been shown to metabolize beta-blockers, impacting their pharmacokinetics and potentially altering blood pressure control [25,118]. These interactions underscore the complexity of drug–microbiome relationships (Figure 3) and highlight the need for personalized approaches in cardiovascular treatment based on individual microbiome profiles.

The oral microbiome significantly impacts the metabolism and effectiveness of cardiovascular medications, often through indirect mechanisms. For instance, *P. gingivalis*, a major player in periodontal disease, is known to exacerbate systemic inflammation. This heightened inflammatory state may decrease the efficacy of antiplatelet drugs such as aspirin and clopidogrel by enhancing the resistance to their anti-inflammatory effects [119,120]. Another example is *Streptococcus mutans*, an important driver of dental caries, which can affect the metabolism of nitrate-based medications [121]. These drugs, used to treat angina, rely on nitrate-to-nitrite conversion in the saliva, a process that may be disrupted by bacterial activity, thereby altering drug performance in blood pressure regulation [121]. Additionally, *F. nucleatum* has been linked to systemic conditions like increased blood viscosity and endothelial dysfunction [122]. Such effects can indirectly diminish the efficacy of anticoagulants like warfarin, potentially leading to a higher risk of thrombosis [123]. These examples demonstrate that oral bacteria not only contribute to local oral health issues but can also influence the pharmacodynamics of cardiovascular medications, making the oral microbiome an essential factor in personalized CVD treatment strategies.

### 5.1. Drug Metabolism and Bioactivation

**Figure 4:**
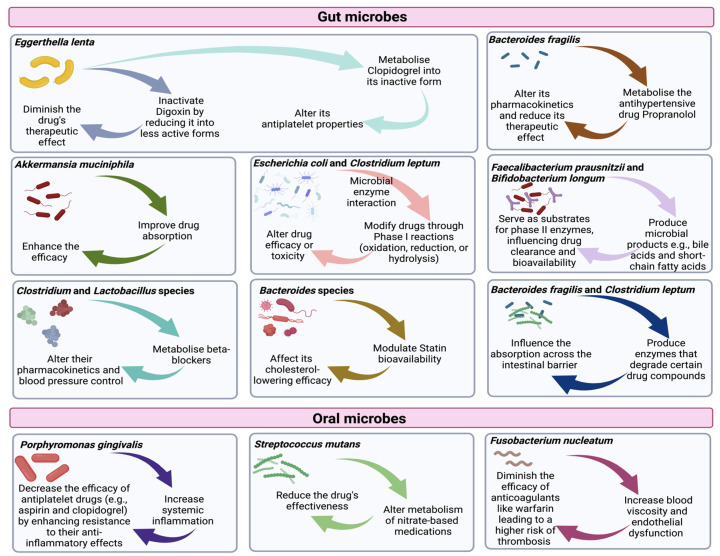
How the oral and gut microbiomes affect CVD drugs’ metabolism. This figure highlights how gut and oral microbes affect the metabolism, efficacy, and absorption of cardiovascular medications. Key interactions involve microbes modifying drugs like digoxin, clopidogrel, and beta-blockers, either enhancing or diminishing their therapeutic effects. The figure also shows the role of microbial metabolites in altering drug bioavailability and systemic inflammation, potentially impacting treatment outcomes.

Drug metabolism and bioactivation refer to the processes through which drugs undergo modification and transformation within the body. These alterations can serve either to facilitate elimination or to allow the drugs to become pharmacologically active [124,125]. Such processes play a crucial role in determining a drug’s efficacy, safety, and duration of action [126,127]. Although drug metabolism primarily occurs in the liver, other organs and tissue types, and also the gut microbiome, contribute to drug metabolism [128,129,130]. Within the gut microbiome, various enzymes capable of metabolizing drugs exist [131,132]. This microbial metabolism has the potential to transform inactive drug compounds into active forms or modify drugs to either enhance or reduce their effectiveness (Figure 4) [133]. For example, the gut microbiome can activate specific prodrugs, such as clopidogrel, into their active forms [134].

Conversely, microbial metabolism can also lead to the inactivation or degradation of certain drugs [135]. Zimmermann et al. (2020) conducted a study highlighting the role of gut microbial metabolism in the inactivation of the antiplatelet drug clopidogrel [136]. Clopidogrel is commonly prescribed to prevent cardiovascular events in patients with atherosclerotic diseases. Their research revealed that specific bacterial species within the gut microbiome, such as *E. lenta*, possess the capability to metabolize clopidogrel into its inactive form [136]. Additionally, the team identified a key bacterial enzyme, β-glucuronidase, responsible for this microbial-mediated metabolism [136].

Phase I and phase II drug metabolism are two distinct processes involved in the biotransformation of drugs within the body. Phase I metabolism involves various enzymes, such as cytochrome P450 (CYP) enzymes, which catalyze reactions such as oxidation, reduction, and hydrolysis [137]. These reactions modify drugs, making them more polar and preparing them for further transformation or excretion. Interestingly, the gut microbiome, particularly certain bacterial species such as *E. coli* and *Clostridium leptum*, also contains enzymes with similar functions. Microbial metabolism in the gut can contribute to the phase I metabolism of drugs, leading to the formation of metabolites that may be more or less active than the parent drug [138,139]. Subsequently, these microbial-derived metabolites can significantly influence the drug’s efficacy or potential toxicity. *Bacteroides fragilis* can metabolize the antihypertensive drug propranolol, altering its pharmacokinetics and potentially reducing its therapeutic effect.

Phase II metabolism encompasses conjugation reactions, wherein drugs or their metabolites bind with endogenous molecules to facilitate their elimination [138]. While phase II metabolism is primarily mediated by host enzymes such as UDP-glucuronosyltransferases (UGTs) and sulfotransferases, the gut microbiome can produce microbial metabolites that may serve as substrates for phase II conjugation reactions [130]. A study conducted by Selwyn et al. (2015) investigated the impact of the gut microbiome on the developmental regulation of drug-processing genes in the liver [140]. Their research demonstrated that the absence of a gut microbiome in germ-free mice resulted in altered expression patterns of drug-processing genes, including phase II conjugation enzymes such as UDP-glucuronosyltransferases (UGTs) [140]. Additionally, microbial products like bile acids and short-chain fatty acids, produced by gut bacteria such as *Faecalibacterium prausnitzii* and *Bifidobacterium longum*, can serve as substrates for phase II enzymes, influencing drug clearance and bioavailability. These microbial contributions can lead to variability in how drugs are metabolized and eliminated, affecting their therapeutic efficacy and potential toxicity. This microbial contribution to phase II metabolism can significantly influence drug clearance and bioavailability.

### 5.2. Drug Absorption and Bioavailability

The gut microbiome plays a role in influencing the absorption and bioavailability of certain drugs. The presence of bacterial species or microbial metabolites can impact the integrity of the intestinal barrier, potentially affecting drug absorption [141]. Additionally, the gut microbiome can influence the expression and functionality of drug transporters responsible for moving drugs across the intestinal wall and into systemic circulation [142]. The term “pharmacokinetics” refers to the study of how drugs are absorbed, distributed, metabolized, and eliminated in the body [143,144]. Drug absorption is a crucial step in the pharmacokinetic process, determining the rate and extent to which a drug enters the systemic circulation and reaches its target site [145]. As many drugs are orally administered, the gut significantly contributes drug absorption. These processes involve several mechanisms, including passive diffusion, active transport, disposition, facilitated diffusion, endocytosis, and pinocytosis.

The gut microbiome can influence drug absorption through various mechanisms, such as gut barrier integrity, engaging in drug–microbiota interactions, and participating in the metabolism of drug precursor metabolism (Figure 4). For example, bacteria such as *B. fragilis* and *Clostridium leptum* can produce enzymes that degrade certain drug compounds, influencing how well they are absorbed across the intestinal barrier [146]. Within the intestinal tract, the gut microbiome can impact the integrity of the intestinal barrier, which consists of epithelial cells connected via tight junctions [147]. Disruptions in the intestinal barrier due to microbial dysbiosis can increase the intestinal permeability, facilitating drug passage and affecting their absorption [148]. Similarly, within the oral cavity, the oral microbiome can modulate the permeability of the oral mucosa, thereby influencing drug absorption [149]. Bacterial metabolites, like short-chain fatty acids (SCFAs), can modulate the permeability of the intestinal barrier by affecting the integrity of the tight junctions between epithelial cells [150,151]. When these junctions are disrupted, as seen in microbial dysbiosis, the intestinal permeability increases, allowing for the enhanced passage of drugs like digoxin, thus altering its absorption and bioavailability [150].

Research shows that statins like simvastatin and atorvastatin can alter the abundance of key gut bacteria, such as *Lactobacillus* and *Bifidobacterium* [73]. These microbes are involved in bile acid metabolism, and their alteration can impact the drug’s efficacy. For example, *Bacteroides thetaiotaomicron* influences the metabolism of bile acids, which in turn affects the solubility and absorption of statins [152]. This interaction can lead to variations in the therapeutic effectiveness of statins, with changes in the gut microbial composition contributing to differences in drug bioavailability between individuals [152].

Similarly, the oral microbiome can also influence drug absorption. *S. mutans*, a bacterium associated with dental caries, is known to alter the oral mucosal environment, potentially affecting the permeability of the mucosa and, thus, the absorption of drugs administered sublingually or buccally, such as nitroglycerin [151]. Moreover, microbial metabolites like SCFAs, produced in the oral cavity, can enhance or inhibit the mucosal barrier’s integrity, impacting the drug’s pharmacokinetics and overall bioavailability [80]. These interactions illustrate how both the gut and oral microbiomes play a critical role in the absorption and efficacy of medications.

## 6. Effects of Specific Cardiovascular Medications on the Microbiome

While the research on the effects of cardiovascular medications on the microbiome is still limited, some studies have provided insights into the potential interactions between CVD medications and the microbiome [33,34,153,154]. Pharmaceuticals commonly prescribed for CVDs, including statins, antiplatelet agents, and ACE inhibitors, have been observed to potentially exert an influence on the composition and functionality of the human microbiome.

### 6.1. Effects on Gut Microbiome

Statins are commonly prescribed medications for the management of high LDL-lipoprotein cholesterol levels in the blood [155] and may alter the composition of the gut microbiome [153,154,155,156]. For example, one human study in China has reported that these medications can decrease the levels of certain bacteria, such as Clostridia, while increasing others, such as Bacteroidetes [154]. In the same study, the gut microbiomes of 202 patients with hyperlipidemia from East China, categorized as either statin-sensitive (SS) responders or statin-resistant (SR) responders, were examined using high-throughput sequencing [154]. The findings of this study revealed that individuals who displayed a positive response to statin treatment tended to have higher biodiversity in their gut microbiome. Furthermore, specific changes in the abundance of certain bacterial genera were observed. The genera *Lactobacillus*, *Eubacterium*, *Faecalibacterium*, and *Bifidobacterium* were found to be increased, while the genus Clostridium showed a decrease in the gut microbiomes of patients with a favorable statin response [154]. In another pre-clinical study, the researchers examined how statin treatment influenced the composition of the gut microbiome in mice [156]. Their results indicated that statins caused changes in both the size and composition of the bile acid pool in the intestine. Bile acids can influence the growth and composition of the gut microbiome, and changes in bile acid levels can create an environment that favors the growth of certain bacteria over others [156]. Human studies have reported similar findings, with statin use associated with changes in gut microbial diversity and composition [153,157]. Certain bacteria, such as *Lactobacillus* species and *Bifidobacterium* species within the gut microbiome, play a role in cholesterol metabolism, and alterations induced by statins may affect the abundance and activity of these microbes, contributing to shifts in the overall composition of the gut microbial community [157].

Beta-blockers are medications that can lower the heart rate and blood pressure by blocking beta receptors in the body. Recent studies suggest that these drugs may also have an impact on the gut microbiome [158,159]. Tabeta et al. (2021) discovered that beta-blocker users showed differences in the alpha diversity of their gut microbiomes compared to non-users among Japanese hospitalized patients (age 66) [160]. Notably, they observed a relative increase in the abundance of the genus *Streptococcus* and order *Lactobacillus* among those taking beta-blockers [160].

### 6.2. Effects on Oral Microbiome

Several studies suggest that the use of statins may have an impact on the composition of the oral microbiome, showing mixed results regarding its influence, both positive and negative [160,161,162]. For instance, research has shown that statins can alter the relative abundance of certain oral bacteria, including reducing the levels of periodontal pathogens associated with gum disease [161]. The results demonstrated that statins effectively inhibited the growth of *Porphyromonas gingivalis*, a specific bacterium, and significantly reduced the overall bacterial population in developing and mature biofilms [161]. Moreover, the specific effects of statins on the oral microbiome may vary depending on factors such as the type of statin, the dosage, and individual variations [162]. Similar to the gut, beta-blockers have also been associated with potential changes in the oral microbial community [163]. For instance, a study by Kgoe et al. (2022) examined individuals with periodontitis and found that the use of beta-blockers was linked to alterations in the composition of the oral microbiome, including changes in the relative abundance of certain bacterial species [163]. More research is needed to comprehensively grasp the significance and consequences of these findings in humans.

## 7. Discussion

Recent studies have illuminated that the oral microbiome, which is intricately linked to periodontal health and dental hygiene, can undergo alterations in response to specific cardiovascular medications, such as statins and beta-blockers (Table 1). These alterations may have systemic implications, as the oral microbiome’s balance has been associated with conditions like endocarditis, which can be exacerbated by oral microbial imbalances.

Moreover, investigations into the gut microbiome have revealed that certain cardiovascular drugs can impact its composition and functioning. Dysbiosis within the gut microbiome, resulting from these medication-induced changes, may influence drug metabolism and absorption, suggesting that the gut microbiome can indirectly influence the effectiveness of these medications in managing cardiovascular diseases.

As we anticipate ongoing research in this domain, it promises to provide a deeper understanding of the intricate relationships between medications, the oral and gut microbiomes, and human health. While the current studies have begun to uncover the impact of certain cardiovascular disease (CVD) medications on these microbiomes, it is important to note that the research in this area is still in its early stages. Only a limited number of CVD medications have been extensively studied, and there remains a significant gap in our knowledge regarding the full spectrum of these interactions.

Future research is needed to expand our understanding and to include a broader range of medications and patient populations. This knowledge is poised to shape the development of more comprehensive and personalized approaches to cardiovascular care in the future. These approaches will consider each individual’s unique microbiome profile, enhancing the treatment outcomes and paving the way for more effective and tailored strategies to combat cardiovascular diseases. Further clinical trials and studies will be essential in validating these findings and integrating them into clinical practice.

This review emphasizes the complex interactions between cardiovascular medications and the human microbiome, but several limitations warrant attention. Much of the current research is based on preclinical studies or small-scale clinical trials, which may restrict the applicability of the findings to broader populations. Additionally, the complexity and uniqueness of each person’s microbiome makes it difficult to standardize the results across diverse patient groups. Human testing for drugs also introduces ethical and logistical challenges, as individual variations in the microbiomes and the responses to medications can complicate data interpretation. Moreover, many studies focus on a narrow range of drugs and microbial species, often neglecting potential interactions with other medications and less-studied microbial communities. To overcome these limitations, we need more extensive, multi-center clinical trials involving diverse populations to gain a thorough understanding of these interactions and to validate the findings for clinical practice.

## 8. Conclusions

In conclusion, the evolving field of research into the interaction between cardiovascular medications and the oral and gut microbiomes in individuals with cardiovascular disease has yielded interesting findings. While these medications are primarily prescribed for their cardiovascular benefits, early evidence highlights their potential to significantly influence the microbial ecosystems within the oral cavity and the gastrointestinal tract. Studies have shown that specific cardiovascular drugs, such as statins and beta-blockers, can cause gut and oral microbial dysbiosis. Additionally, the gut and oral microbiomes can impact the effectiveness of CVD medications, demonstrating a significant bidirectional relationship. This imbalance in the microbial community impacts the metabolism and absorption of these medications, potentially leading to variations in drug responses. A deeper understanding of how these drugs interact with the microbiome could lead to more personalized and effective treatments for cardiovascular disease. Future studies should continue to explore this bidirectional relationship, aiming to develop strategies that mitigate the adverse effects on the microbiome while enhancing drugs’ efficacy.
